# IUC24398-91 Functional and oncological outcomes of robot-assisted radical prostatectomy in obese men: a matched-pair analysis

**DOI:** 10.1093/oncolo/oyaf248.031

**Published:** 2025-08-29

**Authors:** D D Carbin Joseph, S Dranova, H Harrison, D Papanikolou, S Uribe, M Broe, C Adamou, D Whiting, G Frajkoulis, M J A Perry

**Affiliations:** The Stokes Centre for Urology, Royal Surrey County Hospital, Guildford, United Kingdom; The Stokes Centre for Urology, Royal Surrey County Hospital, Guildford, United Kingdom; The Stokes Centre for Urology, Royal Surrey County Hospital, Guildford, United Kingdom; The Stokes Centre for Urology, Royal Surrey County Hospital, Guildford, United Kingdom; The Stokes Centre for Urology, Royal Surrey County Hospital, Guildford, United Kingdom; The Stokes Centre for Urology, Royal Surrey County Hospital, Guildford, United Kingdom; The Stokes Centre for Urology, Royal Surrey County Hospital, Guildford, United Kingdom; The Stokes Centre for Urology, Royal Surrey County Hospital, Guildford, United Kingdom; The Stokes Centre for Urology, Royal Surrey County Hospital, Guildford, United Kingdom; The Stokes Centre for Urology, Royal Surrey County Hospital, Guildford, United Kingdom

## Abstract

**Background:**

Obesity (BMI ≥ 35 kg/m^2^) is a growing global health concern and presents technical challenges during robot-assisted radical prostatectomy (RARP). Despite increasing prevalence, limited data exist on surgical and oncological outcomes of RARP in severely obese patients, especially in the United Kingdom. This study evaluates the safety and efficacy of RARP in this population using a matched-pairs analysis.

**Methods:**

We conducted a retrospective review of 1,273 patients undergoing RARP between January 2018 and June 2021. Forty-three patients with BMI ≥ 35 kg/m^2^ were matched 1:1 with patients having BMI < 35 kg/m^2^ based on PSA, Gleason grade, clinical stage, D’Amico risk, and nerve-sparing status. Perioperative outcomes, 1-year continence rates, and biochemical recurrence (BCR) were assessed. Statistical analyses included Wilcoxon rank-sum and logistic regression (*P* < .05 considered significant).

**Results:**

Both groups were comparable in PSA, tumor stage, grade, and nerve-sparing, though the higher BMI group was younger (*P* < .001). No significant differences were observed in console time (*P* = .20), blood loss (*P* > .90), complications, or BCR at 1-year follow-up (*P* > .90). Continence rates at 1 year were equivalent across both cohorts. Multivariable logistic regression identified age (*P* < .001) and nerve-sparing extent (*P* = .038) as independent predictors of continence recovery; BMI was not a significant factor.

**Conclusions:**

RARP in patients with BMI ≥ 35 kg/m^2^ is safe and yields similar functional and oncological outcomes compared to non-obese counterparts when performed in experienced centers. High BMI alone should not exclude patients from surgical treatment of localized prostate cancer.

